# Spotlight on Cancer Informatics

**Published:** 2007-02-13

**Authors:** Georgios S Stamatakos

**Affiliations:** National Technical University of Athens, Athens, Greece Email gestam@central.ntua.gr

**Q:** What would you say is the primary focus of your research effort (how do you refer to your ‘subarea’)?

**A:** *In Silico* Oncology

**Q:** What do you consider to be the most significant open questions and research challenges in cancer informatics?

**A:** I think that understanding and effectively modeling the *dynamics* of cancer and affected normal tissues at *all* biocomplexity levels by using any efficient combination of mathematical and computer modeling approaches (discrete, continuous, deterministic, stochastic, analytical, numerical, algorithmic etc.) is the fundamental open question and research challenge in *cancer informatics.* Obviously this is a long term target which presupposes success in understanding and modeling every single critical mechanism involved in cancer and affected normal tissue development and treatment response, as well as the subsequent integration of all those modeling modules.

**Figure:**
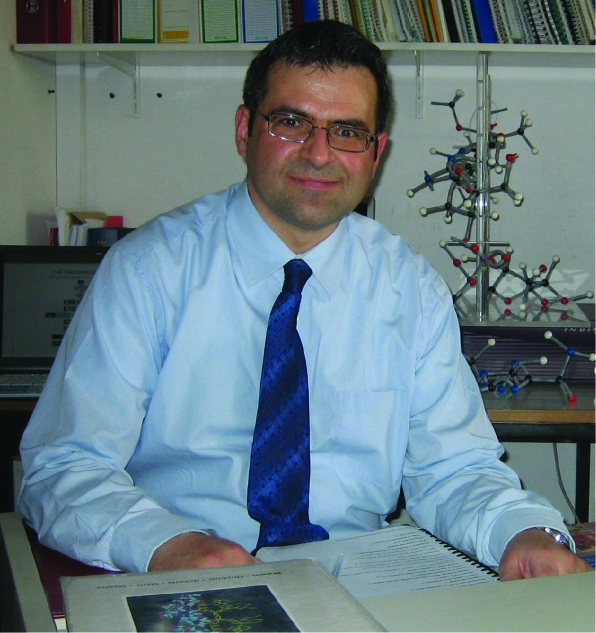


As the demands of such an endeavor are tremendous, I think that a parallelism with the history of Newtonian physics might serve as a source of guidance, inspiration and courage. It has been suggested that cancer epitomizes the entire biology. In this context I think that a title like: “Philosophiae Naturalis Principia Mathematica: Pars II, Materia Vivens” (Mathematical Principles of Natural Philosophy: Part II, Living Matter) might to some extent describe the *collaborative* efforts on a worldwide scale to apply the *analytical* way of thinking on the description of natural phenomena (mechanisms) involving living matter and especially on those related to cancer. Obviously *stochasticity* would be a key player in such an approach. A thorough, *quantitative*, *clinically validated* and *exploitable* understanding of such multi-scale phenomena is expected to dramatically accelerate the achievement of *cancer cure* on a *patient individualized basis* through treatment optimization *in silico* (on the computer). Such an expectation seems to be compatible with the US National Cancer Program’s goal of eliminating the suffering and death due to cancer by 2015.

**Q:** What do you consider to be the most significant developments as a result of research cancer informatics?

**A:** Radiotherapy treatment planning is perhaps the first large scale achievement of cancer informatics. Recent achievements include the design of cancer drugs, the simulation and elucidation of specific tumor growth mechanisms, the modeling of molecular networks involved in cancer etc.

**Q:** Tell us about your collaborative research. How much of your effort is typically focused on helping to provide cancer researchers with clinically significant results?

**A:** I would say that roughly 40% of my effort is focused on helping providing cancer researchers with clinically significant results. Within this frame a careful use of clinical data stemming from clinical trials as well as directly form collaborating hospitals (imaging, histopathological, molecular, historical data) is being made in order to validate, adapt and optimize the simulation models that my group has been developing.

**Q:** What do you consider to be the most pressing challenges or barriers to success in cancer research?

**A:** I consider lack of efficient coordination of the experimental, theoretical and computational research work on a worldwide scale is one of the most pressing barriers to cancer research. Hopefully recent efforts based on grid and other forms of information technology seem to considerably alleviate this problem, but it won’t be enough. IT is important; however, equal funding and effort should be put into the development of sufficiently fine-grained analytical informatics, supporting research on algorithms for cancer modeling, including computationally intensive statistical analyses, understanding information flow about cancer and cancer care as multi-scale phenomena. Past funding priorities in basic research, and now in informatics, have actually starved the development of new insight from mathematics, intensive computing, and statistics, and what we do with the integrated data is, in many ways, fundamentally much more important that how we store and transfer it. The theory in these models must be made to reflect the complexity of cancer, and cancer research and clinical practice must be made ready to be informed by these models. This is especially challenging because we must avoid building IT systems that limit discovery and exploration by hard-wiring a particular knowledge based or paradigm. Ideally, the IT infrastructure would be built with a deep understanding of the intrinsic complexity and multiscale nature of the biological processes involved in cancer occurrence and progression. **In other words apart from a sophisticated infrastructure constructor, *informatics* is called to act - to a certain extent-as the “successor” of classical mathematics i.e. as the descriptive language of hypercomplex natural phenomena such as cancer.** Resolving this paradox requires discourse among open minds, and *Cancer Informatics* is an important start in facilitating such discussions.

**Q:** What do you consider to be the most significant developments as a result of cancer research?

**A:** I think that increase in the rate of curability and life expectancy as well as improvement of the quality of life *for specific cancers* are the most significant *end results* of cancer research. Intermediate results include, for example, development of highly sophisticated technology for radiotherapy treatment planning, computer based design of cancer drugs, modeling and simulation of molecular pathways, tumor growth and response to therapeutic interventions etc.

**Q:** When did you decide to be, or realize that you were, involved primarily in informatics as a research focus?

**A:** I decided to get involved primarily in informatics as a research focus just before the preparation of my diploma thesis at the Department of Electrical and Computer Engineering, National Technical University of Athens.

**Q:** Do you currently conduct research on diseases other than cancer?

**A:** No.

**Q:** Tell us about three or four ‘must-have’ essential informatics computing or research resources that you use on a regular basis developed by someone other than yourself or collaborators. Why are these resources so useful, and why do you consider them essential?

**A:** Visual C++ ™, Matlab ™, AVS ™, CAVE™ Immersive Virtual Reality System

**Q:** What do you think about the development of open access publishing and open access development? How has either changed your perspective on research and development practices?

**A:** I think that open access publishing and open access development have become *sine qua non* necessities in modern *collaborative* scientific research. Two large scale international research projects in which I participate, namely the US NIH *Cancer Integrative Biology Program (Center for the Development of a Virtual Tumor)* and the recently funded European IST project *Advancing Clinicogenomic Trials on Cancer (ACGT)* have adopted the open access development strategy.

**Q:** What books do you think should be required of researchers involved in informatics? In cancer research?

**A:** I think that researchers involved in *both* cancer research and informatics need updated books on 1) general biology including biochemistry 2) pharmacology 3) radiobiology 4) cancer pathology, biology and treatment 5) algorithms and complexity focused on cancer modeling and related issues 6) mathematical analysis (differential equations etc.) 7) statistics 8) general informatics (e.g. computer languages, data bases, image processing, virtual reality, algorithm optimization, grid technologies etc.) 9) other related subjects.

**Q:** What books are on your current reading list?

**A:** A quite large number of books covering all the previously mentioned areas are on my current reading list. Especially, I would like to mention the review book Cancer Bioinformatics: from therapy design to treatment Edited by Sylvia Nagl © 2006 John Wiley & Sons, Ltd (http://eu.wiley.com/WileyCDA/WileyTitle/productCd-0470863048,descCd-tableOfContents.html)

A chapter entitled *Computer Simulation of Tumour Response to Therapy* (by G. S. Stamatakos and N. Uzunoglu) which appears within this book gives a comprehensive outline of some representative efforts concerning both the shaping and the development of the emerging field of *In Silico* Oncology.

**Q:** Do you teach any courses? If so which ones?

**A:** Yes. Within the last years I have taught selected chapters and laboratory classes on Simulation of Physiological Systems as well as full courses on Electromagnetic Fields and Applications.

**Q:** List the historical research figures that you think have most influenced how you think about research? Why are these influences significant?

**A:** I think that the historical figures that have most influenced my way of thinking about research are the following: 1) *Aristotle*, the founder of the science ofbiology, through his extensive zoological descriptions and his detailed and mostly objective observations on the biological phenomena 2) *Isaac Newton*, the founder of classical physics, through his parsimonious (*laconic*) mathematical description of the basic natural phenomena 3) *Gregor Johann Mendel*, the founder of genetics, through his insistent experimentation and the ingenious phenomenological interpretation of his experimental data; still more, through his determination to carry on research despite the unusually unfavorable circumstances he faced during his life.

**Q:** Which research meetings do you attend on a regular basis (please provide URLs)?

**A:**

▪ IEEE EMBS (http://www.ee.cuhk.edu.hk/EMBC05shanghai/),
▪ Drug Discovery Technology(http://www.drugdisc.com/section.asp),
▪ ECO (http://professional.cancerconsultants.com/conference_ecco_2003.aspx),
▪ ASTRO (http://www.astro.org/),
▪ ESTRO (http://www.estroweb.org/estro/index.cfm)

**Q:** Please tell us about your own resource development efforts (limit 3). Which of your computing resources or research papers would you like most people to know about?

**A:** 1) Development of software simulating tumor growth and response to radiotherapeutic schemes 2) Development of software simulating tumor growth and response to chemotherapeutic schemes 3) Development of software simulating the response of normal tissues to radiation therapy (and prospectively chemotherapy).

**Representative key papers:**

G.S. Stamatakos, D.D. Dionysiou, E.I. Zacharaki, N.A. Mouravliansky, K. Nikita, N. Uzunoglu, “*In silico* radiation oncology: combining novel simulation algorithms with current visualization techniques”, *Proceedings of the IEEE,* vol. 90, No11, Nov. 2002. 1764–1777 http://ieeexplore.ieee.org/xpl/freeabs_all.jsp?isnumber=22437&arnumber=1046955&count=11&index=6
D. D. Dionysiou, G. S. Stamatakos, N.K. Uzunoglu, K. S. Nikita, A. Marioli, “A four-dimensional simulation model of tumour response to radiotherapy in vivo: parametric validation considering radiosensitivity, genetic profile and fractionation,” *Journal of Theoretical Biology* 230 (2004) 1–20 [Pubmed Link: http://www.ncbi.nlm.nih.gov/entrez/query.fcgi?cmd=Retrieve&db=pubmed&dopt=Abstract&list_uids=15275995&query_hl=2]
E. I. Zacharaki, G. S. Stamatakos, K.S. Nikita, N. K. Uzunoglu, “Simulating growth dynamics and radiation response of avascular tumour spheroid model validation in the case of an EMT6/Ro multicellular spheroid,” *Computer Methods and Programs in Biomedicine* 2004 76, 193—206. [Pubmed link: http://www.ncbi.nlm.nih.gov/entrez/query.fcgi?cmd=Retrieve&db=pubmed&dopt=Abstract&list_uids=15501506&querv_hl=3 ]
V. P Antipas, G. S Stamatakos, N. K Uzunoglu, D. D Dionysiou, R. G Dale, “ A spatio-temporal simulation model of the response of solid tumours to radiotherapy *in vivo:* parametric validation concerning oxygen enhancement ratio and cell cycle duration,” *Phys. Med. Bid.* 49 (2004) 1485–1504 [Pubmed Link: http://www.ncbi.nlm.nih.gov/entrez/query.fcgi?cmd=Retrieve&db=pubmed&dopt=Abstract&list_uids=15152687&query_hl=14]
G.S. Stamatakos GS, V.P. Antipas VP, N.K. Uzunoglu, “Simulating chemotherapeutic schemes in the individualized treatment context: The paradigm of glioblastoma multiforme treated by temozolomide in vivo.” *Comput Biol Med.* 2005 Oct 2; [Epub ahead of print, Pubmed Link: http://www.ncbi.nlm.nih.gov/entrez/query.fcgi?cmd=Retrieve&db=pubmed&dopt=Abstract&list_uids=16207487&query_hl=14]
G. S. Stamatakos, V.P. Antipas, N. K. Uzunoglu, R. G. Dale, “A four dimensional computer simulation model of the *in vivo* response to radiotherapy of glioblastoma multiforme: studies on the effect of clonogenic cell density.” *British Journal of Radiology,* 2006, vol. 79, 389–400 [http://bjr.birjournals.org/cgi/content/abstract/79/941/389].

**Q:** If you could change three things about how informatics research is conducted, used, perceived, or resourced, what would they be?

**A:** I would promote 1) deeper understanding of the problems to be addressed *before* informatics tools are applied or developed 2) emphasis on the *application driven* informatics development 3) tighter collaboration between all participants involved in informatics research and development.

**Q:** What do you think are the most significant cancer research studies in the last year that have been made possible by advances in informatics?

**A:** Clinical applications of DNA and other molecular irrays, simulation of the dynamics of molecular networks, progress in the modeling of tumor growth and response to therapeutic interventions.

**Q:** Do you have any further thoughts that you might like to share?

**A:** I think that a new era in cancer research is dawning. *Cancer Informatics* is undoubtedly a key player. New challenges include the development of highly specialized algorithms for the simulation of *dynamic* phenomena at all levels of biocomplexity, hyper-high performance hardware, promotion of the open access policy etc. Furthermore, the immense necessities of cancer research are expected to greatly contribute to the progress of *informatics* itself. By analogy with the unparalleled progress achieved in mathematical analysis as a result of the needs of *classical physics* it would be quite reasonable to predict a tremendous impact on the development of *informatics* by the needs of *biology* and *cancer science*, especially if funding priorities are aligned to promote such a synthesis.

